# Screening for depression in medical research: ethical challenges and recommendations

**DOI:** 10.1186/1472-6939-14-4

**Published:** 2013-01-08

**Authors:** Aisling M Sheehan, Hannah McGee

**Affiliations:** 1 Division of Population Health Sciences, Royal College of Surgeons in Ireland, Dublin, Ireland; 2 Faculty of Medicine and Health Sciences, Royal College of Surgeons in Ireland, Dublin, Ireland

**Keywords:** Depression, Screening, Ethics, Research

## Abstract

**Background:**

Due to the important role of depression in major illnesses, screening measures for depression are commonly used in medical research. The protocol for managing participants with positive screens is unclear and raises ethical concerns. The aim of this article is to identify and critically discuss the ethical issues that arise when a positive screen for depression is detected, and offer some guidance on managing these issues.

**Discussion:**

Deciding on whether to report positive screens to healthcare practitioners is both an ethical and a pragmatic dilemma. Evidence suggests that reporting positive depression screens should only be considered in the context of collaborative care. Possible adverse effects, such as the impact of false-positive results, potentially inappropriate labelling, and potentially inappropriate treatment also need to be considered. If possible, the psychometric properties of the selected screening measure should be determined in the target population, and a threshold for depression that minimises the rate of false-positive results should be chosen. It should be clearly communicated to practitioners that screening scores are not diagnostic for depression, and they should be informed about the diagnostic accuracy of the measure. Research participants need to be made aware of the consequences of the detection of high scores on screening measures, and to be fully informed about the implications of the research protocol.

**Summary:**

Further research is needed and the experiences of researchers, participants, and practitioners need to be collated before the value of reporting positive screens for depression can be ascertained. In developing research protocols, the ethical challenges highlighted should be considered. Participants must be agreeable to the agreed protocol and efforts should be made to minimise potentially adverse effects.

## Background

An increasing body of evidence has demonstrated the significant role of depression in major illnesses. A higher incidence of depression than in the general population has been found in many patient groups, for example, type 2 diabetes [1], cancer [2], and Parkinson’s disease [3]. Approximately one in five patients hospitalised for myocardial infarction (MI) meet criteria for depression, which is three times more common than found in community samples [4]. In addition, meta-analyses have concluded that depression is an independent risk factor for coronary heart disease, and that patients with depression are at double the risk of mortality following a MI [4]. Depression is also associated with reduced adherence to medical treatment or behaviour change recommendations, and with higher healthcare costs [5].

Mounting evidence on the role of depression across diseases has resulted in an increased measurement of depression in research. Standardised, self-rating screening questionnaires are commonly used, a selection of which are summarised in Table 1. These screening measures have acceptable psychometric properties and are practical to administer [6]. Although they cannot be used to diagnose depression, high scores indicate a higher severity of symptoms of depression during a specific time period (e.g. the past week) [7].

**Table 1:** Summary of commonly used depression screening measures

**Measure**	**Items**	**Scale**	**Range**	**Recall period**	**Time to complete**
PHQ9 [8]	9	4-point	0-27	Last 2 weeks	< 3 minutes
CES-D [9]	20	4-point	0-60	Past week	10 minutes
GDS-15 [10]	15	Yes or no	0-15	Past week	2-5 minutes
SCL-90-D [11]	16	5-point	16-80	Past 7 days	< 5 minutes
HADS-D [12]	7	4-point	0-21	Past 7 days	< 3 minutes
BDI-II [13]	21	4-point	0-63	Last 2 weeks	5-10 minutes

Abbreviations: BDI-II, Beck Depression Inventory-II; CES-D, Center for Epidemiological Studies Depression Scale; GDS, Geriatric Depression Scale; HADS-D, Hospital Anxiety and Depression Scale, depression subscale; PHQ-9, Patient Health Questionnaire-9; SCL-90-D, depression subscale of the Symptom Checklist 90.

Numerous studies have investigated the optimal cut-off scores at which depression scores are considered significant and are clinically meaningful. A number of statistics are used to examine the diagnostic accuracy of these cut-off scores including sensitivity and specificity. Sensitivity is the proportion of patients correctly identified as having depression and specificity is the proportion of patients correctly identified as not having depression. These scores vary across studies and according to the characteristics of the population group for which the measure is validated [14]. For example, a cut-off score of ≥18 is recommended for the Beck Depression Inventory-II [13] in primary care settings, which has been demonstrated to yield a sensitivity of 94% and a specificity of 92% [15], and a score of ≥16 has been recommended in post MI patients, for which a sensitivity of 88% and a specificity of 92% was found [16].

Unless the sensitivity and specificity rates are both 100%, the cut-off scores overestimate and underestimate levels of depression. A lower cut-off score will increase the sensitivity of the measure and a higher cut-off score will increase the specificity of the measure, thereby minimising the number of false-positives, i.e. those incorrectly identified as depressed. Optimal cut-off scores are generally higher in populations with a high rate of psychiatric disorders compared to the general population [17].

If participants in a research study screen positive for depression according to the chosen cut-off score of the screening measure, the protocol for managing the care of these participants is unclear. It is not common practice for researchers to give details on how positive screens identified within a study are managed. Research Ethics Committees are beginning to require that a response mechanism for high depression screening scores be in place before research can commence. Deciding on a protocol raises a number of ethical issues. This article considers these issues and offers recommendations based on the available evidence and on the practical experience of conducting a research protocol including depression screening.

## Discussion

It is arguable that it may be unethical to ignore potential depression. Depression as a risk factor for mortality has been shown to be comparable in strength to smoking [18]. The Global Burden of Disease (GBD) study quantifies the health effects of more than 100 diseases and injuries and found that in 2004, unipolar depression was the leading cause of disability in middle- and high-income countries [19]. Participants with positive screens for depression could potentially be referred for a more comprehensive evaluation by a professional qualified in diagnosing and managing depression. Guidelines provided by the American Heart Association (AHA) [20], the U.S. Preventive Services Task Force (USPSTF) [21], and the National Institute for Health and Clinical Excellence (NICE) [22] all recommend this protocol. The challenge is that this places a high demand on mental health services and their treatment capacity [23].

An alternative would be to refer participants to primary care where depression is most commonly managed. However, research suggests that the treatment of depression in primary care is inadequate. Resources are limited in primary care and access to psychological interventions is often not available. Hence, antidepressants are the most commonly prescribed treatment, but are often not patients’ preferred choice of treatment [24]. An estimated 20-30% of those identified as depressed in primary care settings receive adequate care and follow-up, and the majority of patients prescribed antidepressants discontinue them soon after initiation [25]. Three systematic reviews [26-28] on the evidence for screening for depression in primary care settings concluded that reporting screening results to primary care practitioners can improve depressive symptoms when there is additional staff providing depression care support. Benefit was not found in the absence of collaborative care or system improvements or supports, such as clinician training, provision of patient educational material, support staff, follow-up visits, and mental health referrals.

The most appropriate approach when research participants screen positive for depression appears to be the reporting of positive depression screens in the context of collaborative care. This involves the collaboration between medical and mental health specialists for optimal disease management [29], and helps to improve diagnostic accuracy and the quality of care [30]. The USPSTF [21] recommends that clinical settings in which screening for depression occurs should have systems in place for accurate diagnosis, effective treatment, and follow-up for depression. However, information on the care available to research participants is not always readily available. This challenge is particularly pertinent for research conducted across multiple sites. Other ethical challenges also need to be considered before the decision is made to report positive screens. There are a number of possible adverse effects including false-positive results, inappropriate labelling, and inappropriate treatment. In addition, reporting positive screens has implications for confidentiality and informed consent.

Reaching thresholds for depression on screening measures does not guarantee meeting the criteria for a diagnosis of depression. It is estimated that 59% of patients screening positive for depression are incorrectly identified as depressed, i.e. they have false-positive results [25]. The psychometric properties of the chosen screening measure therefore need to be carefully considered. These properties have been demonstrated to vary according to patient group, gender, age, and type of depression [31]. Ideally, evidence on the levels of sensitivity and specificity for the screening measure in the target population of the research should be examined, so that appropriate cut-off scores for that population can be chosen. Choosing a cut-off score with low specificity poses the danger of research participants being inappropriately labelled as depressed and subsequently receiving inappropriate treatment. A high specificity of 95% or more is therefore recommended. This would mean that 1 in 20 positive screens would be false-positives. However, information on the diagnostic accuracy of screening measures in particular populations is not always readily available. Further research on optimal cut-off scores is therefore needed.

Research is also needed on the psychological impact of receiving false-positive results for depression. Although this has not been examined for depression screening [27], receiving false-positive results has been shown to cause psychological distress and negatively impact upon health behaviour for other screening programmes, such as mammography screening [32]. In order to minimise any potential harm when referring positive screens, it should be clearly communicated to both participants and medical professionals that high screening scores are not diagnostic for depression, and information on the diagnostic accuracy of the screening measure should be provided. They should be made aware about the possible transient nature of depressive symptoms and the risk of being incorrectly labelled as depressed. Inappropriate treatment based on an inappropriate label is also possible if further diagnostic testing is not conducted. A position statement of the American College of Preventive Medicine [33] states that “[w]ithout proper follow-up, false-positive scores can lead to harmful labelling, unnecessary additional testing, and inappropriate treatment”. The duration of symptoms, the degree of impairment, and co-morbid physical and psychiatric disorders all need to be evaluated before deciding on appropriate treatment [7].

Issues of informed consent and confidentiality also need to be considered. If positive screens will be referred to medical practitioners, participants should be aware of and agreeable to this referral process, according to ethical principles [34]. The participant information leaflet and consent form should highlight this information clearly. Participants’ depression screening results should be confidential, yet they need to be informed that results will be disclosed to their medical team in the event of a positive screen. They should be aware of which members of their medical team will have access to this information and have the right not to consent to this information being disclosed.

## Summary

The debate on the value of reporting positive screens for depression in research participants to medical practitioners is unresolved. Ethically, it is increasingly difficult to ignore high scores on screening measures. However, it is important that the protocol response results in improved outcomes for patients. The most evidence-based approach appears to be the reporting of positive screens in the context of collaborative care. There is currently no evidence to warrant the referral of positive screens in the absence of collaborative care settings. Researchers therefore need to be aware of the care structures available to participants. This is more challenging to determine in large-scale, multi-centre studies. Potential adverse effects and issues of confidentiality and informed consent also need to be reflected on when considering the referral of positive screens to medical practitioners.

Evidence on the psychometric properties for the screening measure in the target population should be ascertained, where possible. The diagnostic accuracy of the measure should be clearly reported to medical practitioners to whom participants are referred. The non-diagnostic nature of the measure should be emphasised. Participants should also be made aware of the potential for false-positives, and the possible transient nature of their depressive symptoms. Prior to recruitment, all participants need to be agreeable to the referral protocol for positive depression screens. Further research is needed to examine the potential adverse effects of referring positive screens, including the psychological impact of receiving false-positive results and potentially inappropriate treatment. The experiences of other researchers need to be collated so that the potential challenges of referring patients, as discussed here, can be anticipated and resolved. In the meantime, every effort should be made to ensure that the potentially adverse effects of referring positive depression screens in research protocols are minimised.

## Competing interests

The authors declare that they have no competing interests.

## Authors’ contributions

Both AS and HMcG contributed to the conception, design, content, and drafting of the article. Both authors read and approved the final version.

## Pre-publication history

The pre-publication history for this paper can be accessed here:

http://www.biomedcentral.com/1472-6939/14/4/prepub
